# Integrated Analysis of Summary Statistics to Identify Pleiotropic Genes and Pathways for the Comorbidity of Schizophrenia and Cardiometabolic Disease

**DOI:** 10.3389/fpsyt.2020.00256

**Published:** 2020-04-17

**Authors:** Hao Liu, Yang Sun, Xinxin Zhang, Shiyang Li, Dong Hu, Lei Xiao, Yanghui Chen, Lin He, Dao Wen Wang

**Affiliations:** ^1^ Division of Cardiology, Department of Internal Medicine, Tongji Hospital, Tongji Medical College, Huazhong University of Science and Technology, Wuhan, China; ^2^ Hubei Key Laboratory of Genetics and Molecular Mechanisms of Cardiological Disorders, Wuhan, China; ^3^ Bio-X Institutes, Key Laboratory for the Genetics of Developmental and Neuropsychiatric Disorders (Ministry of Education), Collaborative Innovation Center for Genetics and Development, Shanghai Mental Health Center, Shanghai Jiaotong University, Shanghai, China

**Keywords:** schizophrenia, cardiometabolic disease, comorbidity, pleiotropic, GWAS, eQTL

## Abstract

Genome-wide association studies (GWAS) have identified abundant risk loci associated with schizophrenia (SCZ), cardiometabolic disease (CMD) including body mass index, coronary artery diseases, type 2 diabetes, low- and high-density lipoprotein, total cholesterol, and triglycerides. Although recent studies have suggested that genetic risk shared between these disorders, the pleiotropic genes and biological pathways shared between them are still vague. Here we integrated comprehensive multi-dimensional data from GWAS, expression quantitative trait loci (eQTL), and gene set database to systematically identify potential pleiotropic genes and biological pathways shared between SCZ and CMD. By integrating the results from different approaches including FUMA, Sherlock, SMR, UTMOST, FOCUS, and DEPICT, we revealed 21 pleiotropic genes that are likely to be shared between SCZ and CMD. These genes include *VRK2*, *SLC39A8*, *NT5C2*, *AMBRA1*, *ARL6IP4*, *OGFOD2*, *PITPNM2*, *CDK2AP1*, *C12orf65*, *ABCB9*, *SETD8*, *MPHOSPH9, FES*, *FURIN*, *INO80E*, *YPEL3*, *MAPK3*, *SREBF1*, *TOM1L2*, *GATAD2A*, and *TM6SF2*. In addition, we also performed the gene-set enrichment analysis using the software of GSA-SNP2 and MAGMA with GWAS summary statistics and identified three biological pathways (MAPK-TRK signaling, growth hormone signaling, and regulation of insulin secretion signaling) shared between them. Our study provides insights into the pleiotropic genes and biological pathways underlying mechanisms for the comorbidity of SCZ and CMD. However, further genetic and functional studies are required to validate the role of these potential pleiotropic genes and pathways in the etiology of the comorbidity of SCZ and CMD, which should provide potential targets for future diagnostics and therapeutics.

## Introduction

Schizophrenia (SCZ) is a serious mental illness, with approximately 10–20 years life expectancy reduced compared with the general population (1). The most common cause of premature death in people with *SCZ* is cardiovascular disease (CVD), which results in three-fold higher mortality and 10 years shorter life expectancy for patients with *SCZ* than the general population (2). While the increased risk of CVD morbidity and mortality in SCZ can be explained by several factors (*e.g.,* smoking, poor diet, and sedentary behavior) (3), it is now established that cardiometabolic disease (CMD) including body mass index (BMI), coronary artery diseases (CAD), type 2 diabetes (T2D), low-density lipoproteins (LDL), high-density lipoproteins (HDL), total cholesterol (TC), and triglycerides (TG), accounts for the majority of the incidence of CVD-related death in schizophrenic patients (4).

Historically, the high prevalence of CMD among schizophrenic patients has been primarily attributed to unhealthy lifestyle factors and the side effects of antipsychotic medications (5). However, recent evidences have suggested that genetic basis and common biological pathways shared between SCZ and CMD (6–8). For example, Andreassen et al. using genetic-pleiotropy-informed methods detected 10 loci associated with both SCZ and CVD risk factors, which include waist-to-hip ratio, systolic blood pressure, BMI, LDL, HDL, and TG (6). So et al. performed polygenic risk scores, linkage disequilibrium score regression, and Mendelian randomization analysis and showed that genetic basis shared between SCZ and BMI, the causal relationship between SCZ and TG, and common biological pathways (e.g., aldosterone synthesis and secretion, neuronal system, and insulin secretion) shared between SCZ and CMD (8). These evidences provide the**foundation for the genetic factors contribute to the comorbidity of SCZ and CMD.

Despite the fact that abundant genetic variants have been reported to be associated with the comorbidity of SCZ and CMD, understanding the functional consequences of genetic variation and identifying the pleiotropic genes and pathways are challenging in human genetics. First, the linkage disequilibrium (LD), a correlation structure**exists across genetic variation of different loci (9). The top associated variant at a locus is often not the causal variant (10). Second, the complexity of gene regulatory. As genetic variants can affect phenotype through distal regulation of gene expression, the nearest gene to the genome-wide association studies (GWAS) top signal is often not the causal gene (10). Additionally, genetic variation affects gene expression in a tissue-specific manner (11). The complexity of LD and gene regulatory hinder the identification of pleiotropic genes and pathways for the comorbidity of SCZ and CMD.

**Figure 1:**
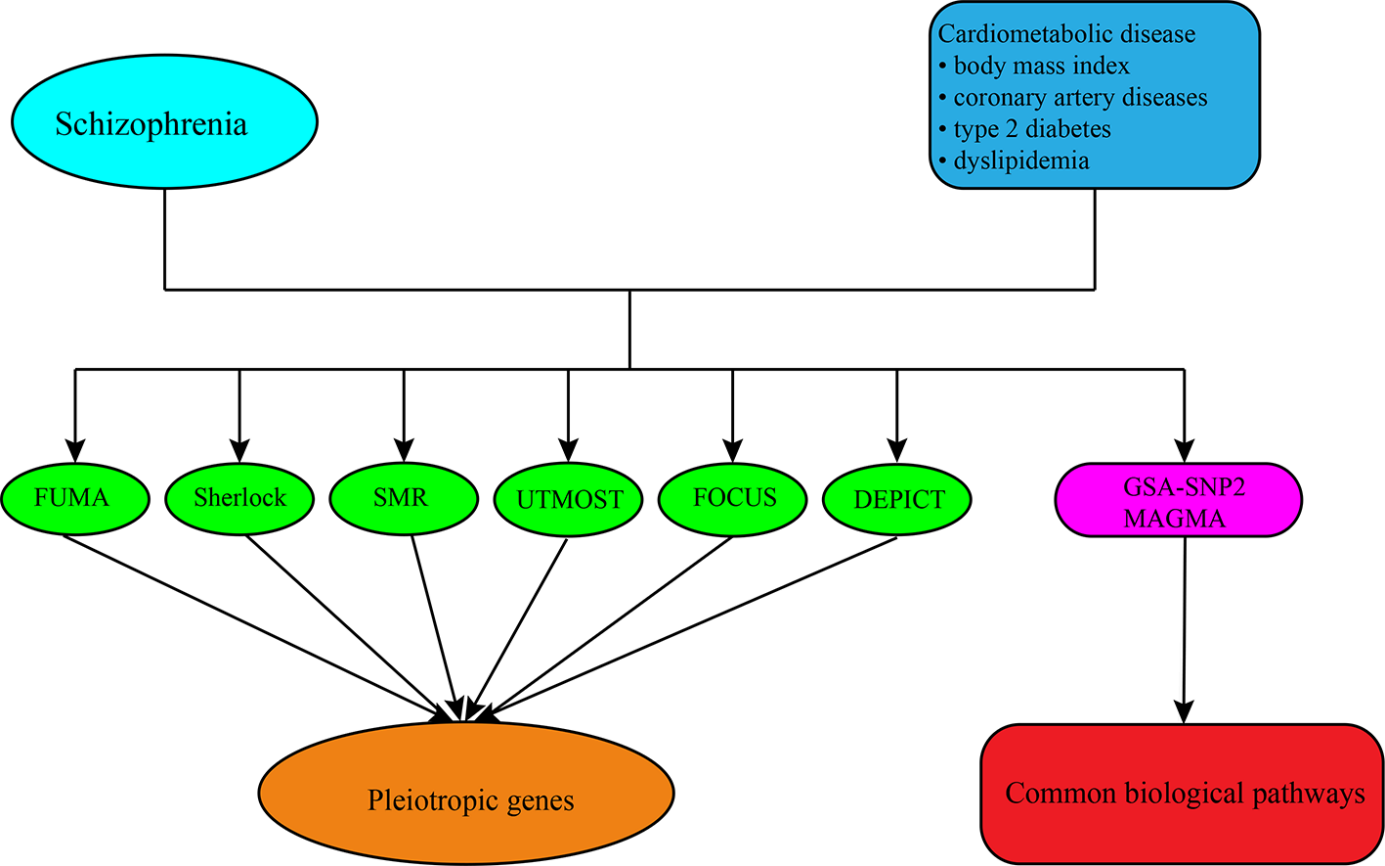
The overall analysis conducted in this study. First, we obtained the summary-level GWAS datasets of SCZ and CMD from public GWAS databases. Then different approaches including FUMA, Sherlock, SMR, UTMOST, FOCUS, and DEPICT were conducted to predicate the candidate genes for them and identified the pleiotropic genes shared between them. Finally, we performed the gene-set enrichment analysis with GWAS summary datasets by the software of GSA-SNP2 and MAGMA to explore the biological pathways shared between them.

In this study, we utilized different approaches and strategies to translate the genetic risk loci into potential candidate genes and pathways for SCZ and CMD, respectively, and then investigated the pleiotropic genes and pathways underlying the comorbidity between them (**Figure 1**). Firstly, we used positional mapping to functionally annotate of traits-associated genetic variants from GWAS summary statistics of SCZ and CMD. We then integrated the GWAS summary statistics of SCZ and CMD, and tissue-specific expression quantitative trait loci (eQTL) data to predicate the causal genes for SCZ and CMD. Finally, we performed gene-set enrichment analysis with GWAS summary statistics to identify the potential biological pathways for SCZ and CMD. This landscape of potential pleiotropic genes and biological pathways will help us to understand clearly the increased risk of CVD morbidity and mortality in SCZ.

## Materials and Methods

### GWAS Summary Datasets

The GWAS summary statistics analysis included in this study were obtained from the following publicly available databases:

- The GWAS data of SCZ was obtained from Psychiatric Genomics Consortium (PGC) (12), which systematically meta-analyzed of the genome-wide genotypes from 49 independent samples (46 of European and 3 of Asian ancestry, including 35,476 SCZ cases and 46,839 controls). Genotype data was processed by the PGC using unified quality control procedures followed by imputation of SNPs and insertion-deletions using the 1000 Genomes Project reference panel. Around 9.5 million variants after quality control were included in the dataset and used in this study.
- The largest-scale GWAS meta-analysis summary data of BMI was performed by Genetic Investigation of ANthropometric Traits (GIANT) (13), which conducted with a total sample of 322,154 individuals of European descent. This GWAS examined the phenotype of BMI as determined from measured or self-reported weight and height, and identified 77 loci reaching genome-wide significance (P < 5 × 10^−8^).
- The summary data of genetic variants associated with CAD was performed by the Coronary ARtery DIsease Genome wide Replication and Meta-analysis plus The Coronary Artery Disease Genetics (CARDIoGRAMplusC4D) Consortium (14), which assembled 60,801 cases and 123,504 controls from 48 studies. The majority (77%) of the participants were of European ancestry; 13% and 6% were of South Asian and East Asian ancestry, respectively, with smaller samples of Hispanic and African Americans. The results of association analysis from an additive model and a recessive model were used in this study.
- Genetic variations associated with T2D were obtained from DIAbetes Genetics Replication And Meta analysis (DIAGRAM) Consortium (15). This study is a meta-analysis from 32 GWAS, including 898,130 individuals (74,124 cases and 824,006 controls) of European ancestry. More than 200 loci reaching genome-wide significance (P < 5 × 10^−8^) in the BMI-unadjusted analysis and 152 loci BMI-adjusted analysis. The GWAS summary statistics with BMI adjustment was considered for our analyses.
- The GWAS summary statistics of dyslipidemia were accessed from the Global Lipids Genetics Consortium (GLGC) (16), which provides the meta-analysis results on four phenotypes: HDL, LDL, TC, and TG. These results are based on GWAS results from 46 cohorts comprising of more than 100,000 individuals of European ancestry (N_HDL_ = 99,900, N_LDL_ = 95,454, N_TC_ = 100,184, and N_TG_ = 96,598).

All GWAS summary statistics used in this study are based on the hg19 human assembly and rsIDs were mapped to dbSNP build 151 using MySQL local database if necessary. We excluded the genetic variants in extended major histocompatibility complex (MHC) region (chr6:25–35MB), due to the complexity of haplotype and LD structure. The major samples in the GWAS summary datasets came from populations of European ancestry. The GWAS summary datasets used in this study were downloaded from publicly available resources listed in **Supplementary Table S1**. More detailed information about sample recruitment and diagnosis, genotyping, quality control, and statistical analysis can be found in the original paper (12–16).

### eQTL Datasets

To evaluate the possible effect of genetic variants on transcriptional activity, we applied different eQTL datasets including Schadt et al. (17), Myers et al. (18), Westra et al. (19), Lloyd-Jones et al. (20), Qi et al. (21), Battle et al. (22), and V7 release summary data of the Genotype-Tissue Expression (GTEx V7) project (23). Concisely, Schadt et al. profiled more than 39,000 transcripts and genotyped 782,476 unique single nucleotide polymorphisms (SNPs) in 427 human liver samples of Caucasian individuals to characterize the genetic architecture of gene expression in human liver (17). Myers et al. carried out whole-genome genotyping and expression analysis on a series of 193 neuropathologically normal human brain cortex samples from the individuals of European descent (18). Westra et al. performed a meta-analysis of eQTL in non-transformed peripheral blood from 5,311 samples with replication in 2,775 individuals (19). Lloyd-Jones et al. analyzed the mRNA levels for 36,778 transcript expression traits to investigate the genetic architecture of gene expression and degree of missing heritability for gene expression in peripheral blood in 2,765 European individuals (20). Qi et al. meta-analysed cis- eQTL between brain and blood to identify putative functional genes for brain-related complex traits and diseases (21). Battle et al. sequenced RNA from whole blood of 922 genotyped the European ancestry individuals from the Depression Genes and Networks cohort for understanding the consequences of regulatory variation in the human genome (22). The GTEx V7 was established to characterize human transcriptomes and has created a reference resource of gene expression levels from non-diseased tissues, including genotype, gene expression, and histological data for 449 human donors across 44 tissues (23). More details about sample description, genotyping, expression quantification, and statistical analyses can be found in the corresponding original paper (17–23).

Considering that genetic variants may affect gene expression in a tissue-specific manner, we used brain and whole blood eQTL for SCZ; subcutaneous adipose, visceral omentum adipose and whole blood eQTL for BMI; left ventricle, atrial appendage, aorta artery, coronary artery, tibial artery, subcutaneous adipose, visceral omentum adipose, liver, and whole blood eQTL for CAD; subcutaneous adipose, visceral omentum adipose, skeletal muscle, liver, pancreas, and whole blood eQTL for T2D; subcutaneous adipose, visceral omentum adipose, liver, and whole blood eQTL for HDL, LDL, TG, and TC.

### Identifying Causal Genes Using Positional Mapping (FUMA)

Functional annotation of genetic variants from GWAS summary statistics was performed using FUMA (24), which incorporates 18 biological data repositories to process GWAS summary statistics. In particular, positional mapping in FUMA was performed by the ANNOVAR annotations of specifying the maximum distance between SNPs and genes, and using Combined Annotation Dependent Depletion (CADD) scores (25) to predict the functional consequences of SNPs on genes. The CADD scores predict how deleterious the effect of an SNP is likely to be for a protein structure or function, with higher scores referring to higher deleteriousness. In this method, we chose the default distance 10kb as the maximum distance, and performed SNPs filtering based on CADD score. The threshold for significance is CADD scores ≥ 12.37 with SNP P-value ≤ 5×10^−8^.

### Integration of GWAS and eQTL Datasets (Sherlock)

Considering that genetic variants may affect the disease through regulation of gene expression, we applied the method named Sherlock (26) to integrate GWAS and eQTL data with the aim to identify causal genes for diseases. Its underlying principle is that any genetic variants perturbs expression levels of risk genes is also likely to influence the risk of disease. Sherlock uses a Bayesian model and the information of SNPs in GWAS and eQTL data to calculate the SNP-level Bayes factor for estimating the association of the SNPs with the expression of gene and the disease, respectively. For the SNPs overlap between eQTL for a gene and the significant SNPs loci associated with the disease, it is straightforward to combine the SNP-level Bayes factor to obtain the Bayes factor for the gene and yielding a single per-gene score to test whether the expression change of this gene has any impact on the risk of disease. Statistical significance was determined using a Bonferroni corrected with P-value < 0.05/the total number of genes.

### Integration of GWAS and eQTL Datasets (SMR)

Summary databased Mendelian randomization (SMR) (27) was used to predict causal genes by integrating the summary-level data from GWAS and data from eQTL studies. The principle of SMR analysis is to use a genetic variant as an instrumental variable to test for the causative effect of the gene expression (the exposure) on the phenotype of interest. The method including two tests: SMR test and heterogeneity in dependent instruments (HEIDI) test. SMR uses the simulation analysis to evaluate the effect of genetic variant on gene expression, genetic variant on phenotype, and gene expression on phenotype, respectively (SMR test). To test whether gene expression and phenotype are affected by the same causative variant, it uses multiple SNPs in a cis-eQTL region to distinguish pleiotropy from linkage (HEIDI test). The gene is considered to be plausible causal gene if pass the SMR (Bonferroni corrected P-value < 0.05) and HEIDI tests (P-value ≥ 0.05).

### Predicting Causal Genes Using Tissue Expression Data (UTMOST)

In contrast to Sherlock and SMR, unified test for molecular signatures (UTMOST) (28) is a powerful approach to studying the genetic architecture of complex traits by using multi-task learning method to jointly impute gene expression in tissues. Briefly, the UTMOST framework includes three main steps. First, it trains a cross-tissue expression imputation model by using the genotype information and matched expression data. Next, it tests the association between the trait of interest and imputed gene expression in each tissue. Finally, a cross-tissue test is performed for each gene to summarize single-tissue association statistics into a powerful metric that quantifies the overall gene–trait association. Statistical significance was determined using a Bonferroni corrected with P-value < 0.05/the total number of genes.

### Predicting Causal Genes Using Expression Weights Data (FOCUS)

To identify potential causal genes involved in complex traits and diseases, we apply the approach of fine-mapping of causal gene sets (FOCUS) (29). This approach is a probabilistic framework that models correlation among transcriptome-wide association study signals to assign a probability for every gene in the risk region to explain the effect of SNPs on a trait. By integrating the GWAS summary data, expression prediction weights (as estimated from eQTL reference panels), and LD among all SNPs in the risk region, it identifies causal gene to be included in a 90%-credible set and give a posterior probability (PIP) to for estimating the causality in relevant tissue types. In this work, we used the recommend eQTL reference panel weight database, which combines GTEx weights with the Metabolic Syndrome in Men study (adipose, n = 563) (30, 31), the Netherlands Twins Registry (NTR; blood, n = 1,247) (32), the Young Finns Study (YFS; blood, n = 1,264) (33, 34), and the CommonMind Consortium (dorsolateral prefrontal cortex, n = 452) (35) weights into a single usable database for FOCUS. The setting of significance threshold is the genes in a 90%-credible set with PIP ≥ 0.5.

### Identifying Causal Genes Using Gene Prioritization Analysis (DEPICT)

We used Data-driven Expression Prioritized Integration for Complex Traits (DEPICT) (36) to prioritize genes at associated loci based on predicted gene functions. Briefly, DEPICT prioritizes genes based on the assumption that truly associated genes should share functional annotations. By using co-regulation data from 77,840 microarrays and publicly available datasets, DEPICT accurately predicts gene function and generated 14,461 “reconstituted” gene sets. Integrating these precomputed gene functions and the GWAS summary data, DEPICT prioritizes genes that share predicted functions with genes from the other associated loci more often than expected by chance. As it has been widely used in previous studies for gene prioritization of SCZ (37, 38) and CMD (39–41), we included their results into our study. We only use this method to prioritizes genes for TC. We chose P-value < 1.0 × 10^−5^ as the GWAS significance threshold, which was recommended by the developers of the DEPICT software. The Benjamini–Hochberg procedure with a threshold of a false discovery rate (FDR) < 0.05 was regarded with statistical significance.

### Pathway Enrichment Analyses

KEGG pathway enrichment analyses were carried out using clusterProfiler package (42) as implemented in R. The significance P-values of the KEGG pathways were corrected by the Benjamini–Hochberg procedure with FDR < 0.05.

### Gene-Set Enrichment Analysis

In order to explore the biological pathways shared between SCZ and CMD, enriched pathways were identified for each trait using GSA-SNP2 software (43), which only requires the P-values of the SNPs in GWAS data and retains SNPs with 20 kb upstream or downstream of a gene. In this study, we used gene set databases of canonical pathways, KEGG, BioCarta and Reactome, which were downloaded from the Molecular Signatures Database (MSigDB) in GSEA (http://software.broadinstitute.org/gsea/msigdb/index.jsp). The Benjamini–Hochberg method is used for the multiple testing correction. The minimum P-values of the pathway was chosen, and the P-values of pathways with FDR < 0.05 were regarded as statistical significance.

To validate the significant finding, the common pathways identified by GSA-SNP2 were investigated by MAGMA (44) with the same parameter settings (retaining SNPs with 20 kb upstream or downstream of a gene). Unlike GSA-SNP2, we used a nominal P-value threshold of P < 0.05.

## Results

To reveal the potential candidate gene for SCZ and CMD, we utilized six different approaches including FUMA, Sherlock, SMR, UTMOST, FOCUS, and DEPICT. To estimate the LD structure, we used the reference data from the European population of 1000 Genomes Project phase 3 (45). All analyses were carried out using the default parameters recommended by the developers if not mentioned in the methods section. A gene may represent a potential candidate gene if it is predicted by two or more than two approaches.

### Candidate Genes Identified for SCZ

Functional annotation of the GWAS summary statistics of SCZ was performed using positional mapping in FUMA, and 110 causal genes associated with SCZ was identified (**Supplementary Table S2**). Through integrating the genetic variations associated with SCZ and tissue-specific eQTL data from brain and whole blood, Sherlock, SMR, UTMOST, and FOCUS identified 198, 50, 236, and 109 causal genes associated with SCZ, respectively (**Supplementary Table S2**). In addition, causal genes prioritized by DEPICT were obtained from Pers et al. (37) and Li et al. (38), which predicted 106 causal genes for SCZ (**Supplementary Table S2**). In total, we identified 553 causal genes whose expression level change may contribute to SCZ risk. KEGG pathway enrichment analysis showed that these causal genes were enriched in dopaminergic synapse (corrected P = 3.3×10^−2^) and adrenergic signaling in cardiomyocytes (corrected P = 4.3×10^−2^) (**Supplementary Table S10**).

Through integrating these causal genes predicted by the six different approaches, we identified 150 potential candidate genes associated with SCZ (**Supplementary Table S2**). Among the 150 genes, only *ABCB9* was predicted by all approaches, which has been reported to be correlated with the risk of SCZ (46). There are seven genes (*ARL6IP4*, *C2orf47*, *GATAD2A*, *GNL3*, *NT5C2*, *PCCB*, and *SNX19*) predicted by five approaches, two genes (*C2orf47* and *PCCB*) of which have not been reported in the literature yet.

### Candidate Genes Identified for BMI

Through integrating the results from FUMA, Sherlock, SMR, UTMOST, and FOCUS, and causal genes predicated by DEPICT for BMI obtained from Võsa et al. (41), we identified 654 causal genes linked to BMI (**Supplementary Table S3**). KEGG pathway enrichment analysis showed that these causal genes were also enriched in dopaminergic synapse (corrected P = 6.8×10^−4^) and adrenergic signaling in cardiomyocytes (corrected P = 2.5×10^−4^) (**Supplementary Table S10**), which were identified to be associated with SCZ.

Among the 654 causal genes, most of them are only predicted by DEPICT, and 79 potential candidate genes were identified (**Supplementary Table S3**). Ten genes (*ZNF668*, *NEGR1*, *KAT8*, *SH2B1*, *BCKDK*, *POC5*, *MAP2K5*, *C18orf8*, *NPC1*, and *C1QTNF4*) were at least predicted by four different approaches, thus represent the most promising candidate genes for BMI. The ten promising candidate genes include six genes (*NEGR1*, *KAT8*, *POC5*, *MAP2K5*, *C18orf8*, and *C1QTNF4*) which were previously reported as causal genes in the original study (13), two genes (*SH2B1* and *NPC1*) have been reported to be associated with BMI by other studies (47, 48), and two genes (*ZNF668* and *BCKDK*) were novel.

### Candidate Genes Identified for CAD

Using the GWAS summary statistics of CAD, we identified 273 causal genes associated with CAD. In detail, FUMA, Sherlock, SMR, UTMOST, and FOCUS identified 41, 73, 85, and 48 causal genes associated with CAD, respectively (**Supplementary Table S4**). Causal genes predicated by DEPICT for CAD obtained from Võsa et al. (41), which predicted 123 causal genes for CAD. Overall, 53 potential candidate genes were identified to be associated with CAD (**Supplementary Table S4**), and 13 candidate genes (*CARF*, *FAM177B*, *GGCX*, *FAM117B*, *TDRD10*, *SWAP70*, *SUSD2*, *RP1-257A7.5*, *VAMP5*, *SPECC1L*, *RGL3*, *KANK2*, *SLC22A1*) can be considered as novel genes.

### Candidate Genes Identified for T2D

Using different approaches (including FUMA, Sherlock, SMR, UTMOST, and FOCUS) to prioritize the causal genes for T2D, we identified 190, 188, 63, 327, and 178 causal genes associated with T2D, respectively (**Supplementary Table S5**). Causal genes predicated by DEPICT for T2D obtained from Scott et al. (40), which predicted 29 causal genes for T2D. Through integrating the results from these approaches, 171 potential candidate genes were identified to be associated with T2D (**Supplementary Table S5**). Among the 171 potential candidate genes, there are eight genes (*ABCB9*, *PABPC4*, *ITFG3*, *ANK1*, *CALR*, *CEP68*, *GCDH*, and *ZZEF1*) predicted by five approaches, five genes (*PABPC4*, *ITFG3*, *CEP68*, *GCDH*, and *ZZEF1*) of which have not been reported in the literature yet.

### Candidate Genes Identified for Dyslipidemia

Through integrating the results from FUMA, Sherlock, SMR, UTMOST, and FOCUS, and causal genes predicated by DEPICT for HDL, LDL, and TG obtained from Bentley et al. (39), we identified 104, 74, 101, and 71 potential candidate gene associated with HDL, LDL, TC, and TG, respectively (**Supplementary Tables S6–9**). Among these potential candidate genes, there are six genes (*CETP*, *APOB*, *TMEM258*, *FADS2*, *FADS1*, and *PVRL2*) associated with all phenotypes of dyslipidemia. Similar to our results, all of the six genes were previously reported to be associated with at least one phenotype of dyslipidemia. Overall, 245 potential candidate genes were identified to be associated with dyslipidemia.

Similar to our results, genetic variants in *NT5C2* were previously reported to be associated with the comorbidity of SCZ and BMI (6), and genetic variants in or around *MPHOSPH9* were reported to be increased risk of T2D in SCZ (49). The remaining genes are considered as novel genes associated with the comorbidity of SCZ and CMD.

### Pleiotropic Genes Identified for the Comorbidity of SCZ and CMD

**Figure 2:**
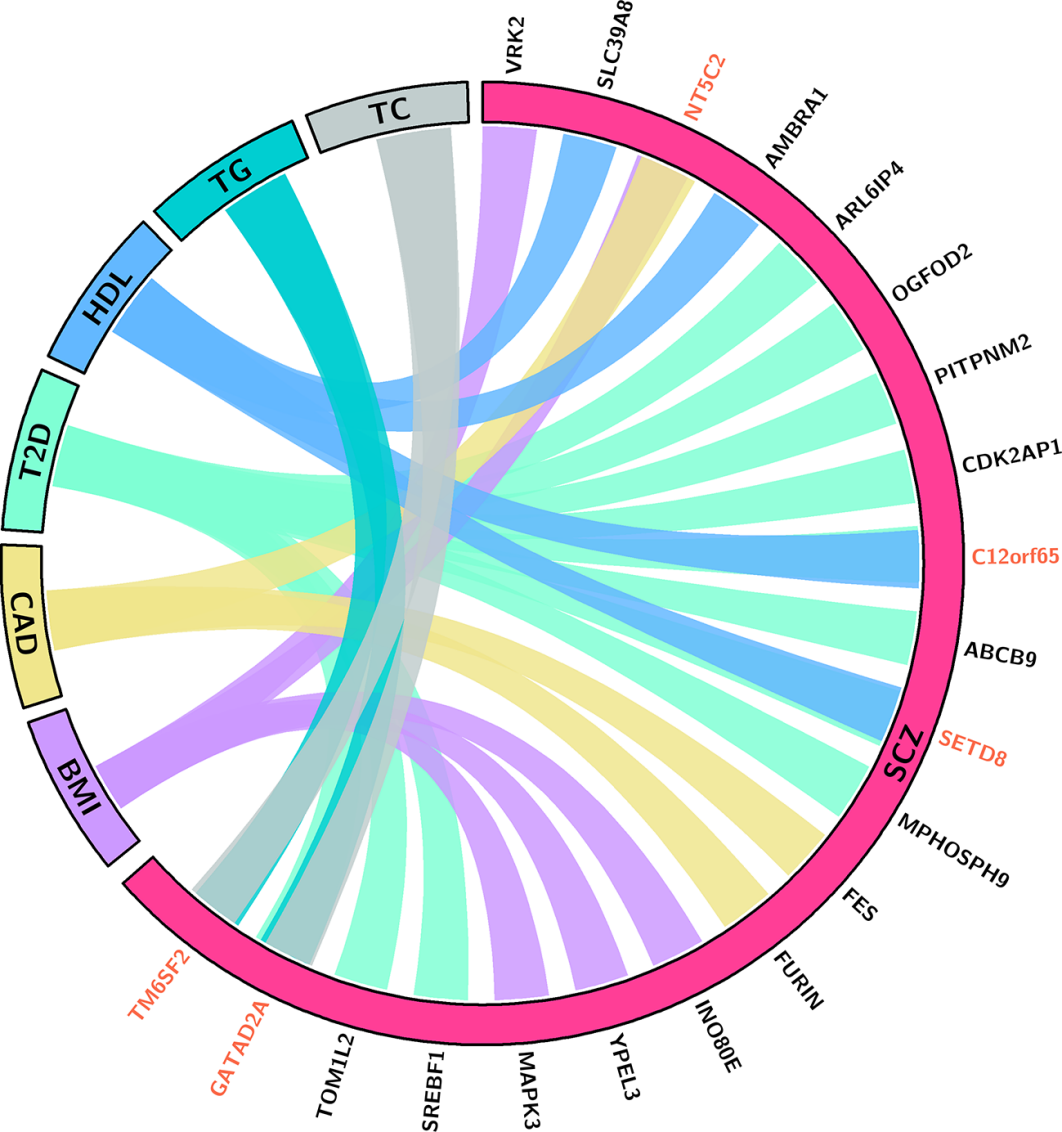
This chord (50) diagram depicts the potential pleiotropic genes shared between SCZ and CMD. Connections indicate that the pleiotropic genes for SCZ and specific phenotype of CMD. Red indicates the gene is associated with SCZ and multiple phenotypes of CMD. BMI, body mass index; CAD, coronary artery diseases; T2D, type 2 diabetes; HDL, high-density lipoproteins; TC, total cholesterol; TG, triglycerides.

Through integrating the potential candidate genes identified for SCZ and CMD, we identified 21 potential pleiotropic genes shared between them (**Figure 2**, **Table 1**). Specifically, *VRK2*, *NT5C2*, *INO80E*, *YPEL3*, and *MAPK3* are the common candidate genes of SCZ and BMI; *NT5C2*, *FES*, and *FURIN* are the candidate genes for both SCZ and CAD; *ARL6IP4*, *OGFOD2*, *PITPNM2*, *CDK2AP1*, *C12orf65*, *ABCB9*, *SETD8*, *MPHOSPH9*, *SREBF1*, *TOM1L2*, and *GATAD2A* are associated with the comorbidity of SCZ and T2D; *SLC39A8*, *AMBRA1*, *C12orf65*, and *SETD8* are associated with the comorbidity of SCZ and HDL; *GATAD2A* and *TM6SF2* are the common candidate genes of SCZ, TC, and TG.

**Table 1:** The potential pleiotropic genes shared between SCZ and CMD are identified in this study.

Gene symbol	Chromosome and position	Diseases (prediction methods)
*VRK2*	ch2: 257907651-58164001	SCZ (FUMA/UTMOST), BMI (UTMOST/DEPICT)
*SLC39A8*	ch4:102251041-102345498	SCZ (FUMA/FOCUS), HDL (FUMA/Sherlock)
*NT5C2*	ch10: 103088017-103193306	SCZ (FUMA/Sherlock/SMR/UTMOST/FOCUS), BMI (Sherlock/FUMA), CAD (Sherlock/SMR/UTMOST)
*AMBRA1*	ch11: 46396412-46594069	SCZ (FUMA/DEPICT), HDL (UTMOST/DEPICT)
*ARL6IP4*	ch12: 122980060-122982913	SCZ (FUMA/Sherlock/SMR/UTMOST/FOCUS), T2D (FUMA/Sherlock)
*OGFOD2*	ch12: 122974703-122980041	SCZ (FUMA/UTMOST/FOCUS), T2D (FUMA/UTMOST)
*PITPNM2*	ch12: 122983480-123150015	SCZ (FUMA/Sherlock/SMR/FOCUS/DEPICT), T2D (FUMA/FOCUS)
*CDK2AP1*	ch12: 123260970-123272316	SCZ (Sherlock/UTMOST), T2D (FUMA/Sherlock/UTMOST)
*C12orf65*	ch12: 123233297-123257959	SCZ (FUMA/UTMOST), T2D (FUMA/Sherlock/UTMOST), HDL (Sherlock/DEPICT)
*ABCB9*	ch12: 122917324-122975160	SCZ (FUMA/Sherlock/SMR/UTMOST/FOCUS/DEPICT), T2D (FUMA/Sherlock/SMR/UTMOST/FOCUS)
*SETD8*	ch12: 123384116-123409356	SCZ (FUMA/Sherlock/UTMOST/DEPICT), T2D (Sherlock/UTMOST/DEPICT), HDL (UTMOST/FOCUS/DEPICT)
*MPHOSPH9*	ch12: 123152324-123244014	SCZ (FUMA/UTMOST), T2D (FUMA/SMR/UTMOST/FOCUS)
*FES*	ch15: 90884421-90895776	SCZ (FUMA/Sherlock/SMR/UTMOST), CAD (Sherlock/SMR/UTMOST/DEPICT)
*FURIN*	ch15: 90868592-90883458	SCZ (FUMA/SMR/UTMOST/FOCUS), CAD (SMR/DEPICT)
*INO80E*	ch16: 29995690-30005794	SCZ (FUMA/Sherlock/UTMOST/FOCUS/DEPICT), BMI (Sherlock/UTMOST/DEPICT)
*YPEL3*	ch16: 30092314-30096216	SCZ (Sherlock/UTMOST/DEPICT), BMI (Sherlock/DEPICT)
*MAPK3*	ch16: 30114105-30123309	SCZ (Sherlock/SMR/UTMOST/FOCUS/DEPICT), BMI (Sherlock/DEPICT)
*SREBF1*	ch17: 17811349-17837017	SCZ (Sherlock/SMR/UTMOST/FOCUS), T2D (SMR/UTMOST/FOCUS)
*TOM1L2*	ch17: 17843508-17972470	SCZ (Sherlock/UTMOST/FOCUS/DEPICT), T2D (SMR/UTMOST/FOCUS)
*GATAD2A*	ch19: 19385803-19508932	SCZ (FUMA/Sherlock/SMR/UTMOST/FOCUS), T2D (FUMA/Sherlock/UTMOST/DEPICT), TC (Sherlock/UTMOST), TG (Sherlock/UTMOST)
*TM6SF2*	ch19: 19264365-19273265	SCZ (Sherlock/FOCUS), TC (Sherlock/DEPICT), TG (Sherlock/DEPICT)

SCZ, schizophrenia; CMD, cardiometabolic disease; BMI, body mass index; CAD, coronary artery diseases; T2D, type 2 diabetes; HDL, high-density lipoproteins; TC, total cholesterol; TG, triglycerides.

### Common Pathway Identified for the Comorbidity of SCZ and CMD

We conducted KEGG pathway enrichment analysis for the causal genes of SCZ and CMD, however, there is no significant pathway enrichment shared between SCZ and CMD. To further explore whether there are some pathogenic pathways shared between SCZ and CMD, we performed gene set enrichment analysis using GSA-SNP2 with GWAS summary datasets. The results show that there are nine pathways shared between SCZ and CMD (**Table 2**, **Supplementary Table S11**). Among these significant pathways, there are two pathways including CXCR4 pathway and growth hormone pathway shared between SCZ and three phenotypes of CMD.

**Table 2:** The potential biological pathways shared between SCZ and CMD are identified in this study.

Pathway	GSA-SNP2							MAGMA		
	BMI	CAD	T2D	LDL	TC	TG	SCZ	BMI	T2D	SCZ
M13494	0.377	**3.49E−03**	0.679	0.295	0.329	0.652	**0.044**	0.177	0.639	**0.02**
M882	**0.018**	**0.038**	0.134	**0.018**	0.056	0.585	**0.041**	0.148	0.134	0.258
M16811	**0.042**	0.264	1.0	**0.043**	0.069	0.193	**6.19E−03**	0.411	**0.018**	**3.24E−03**
M9043	0.175	0.269	**7.36E−03**	**1.22E−03**	**8.92E−03**	0.246	**0.044**	0.803	**0.012**	**0.018**
M255	0.081	**0.013**	**4.4E−03**	0.862	0.487	0.898	**0.04**	0.103	**3.71E−05**	0.2358
M270	**6.76E−04**	0.182	1.0	0.771	0.719	0.898	**0.013**	**6.54E−03**	0.096	**0.022**
M756	**6.05E−03**	0.948	1.0	0.833	0.799	**0.027**	**0.02**	0.117	0.714	0.069
M1921	**0.011**	0.540	**1.04E−04**	0.873	0.799	0.769	**0.036**	0.08	**5.01E−04**	**0.041**
M910	**0.018**	0.220	0.768	0.777	0.763	0.898	**0.036**	0.141	0.223	0.065

The Black body indicates the significance of each approach for diseases. M13494, biopeptides pathway; M882, CXCR4 pathway; M16811, ERK5 pathway; M9043, growth hormone signaling; M255, PID-HIF1 pathway; M270, MAPK-TRK pathway; M756, peptide hormone biosynthesis; M1921, regulation of insulin secretion; M910, synthesis, secretion, and inactivation of glucose-dependent insulinotropic polypeptide; LDL, low-density lipoproteins.

To further validate the significant finding, the common pathways identified by GSA-SNP2 were investigated by MAGMA. However, only one pathway (MAPK-TRK pathway) shared between SCZ and BMI, and two pathways including growth hormone signaling and regulation of insulin secretion signaling shared between SCZ and T2D were confirmed by MAGMA (**Table 2**, **Supplementary Table S12**). Among the three pathways, regulation of insulin secretion signaling was previously reported to be associated with the comorbidity of SCZ and CMD (8). The other pathways can be considered as novel.

## Discussion

As the key risk factor for CVD, CMD was getting more prevalent among patients with SCZ which is chiefly responsible for increasing risk of CVD morbidity and mortality in SCZ. Although unhealthy lifestyle factors and the side effects of antipsychotic medications have been primarily attributed to the high prevalence of CMD in SCZ, shared genetics between SCZ and CMD might also be of importance. Previous studies have shown that both SCZ and CMD are high heritable and polygenic (51, 52). Recent studies have identified numbers of risk loci that are associated with the comorbidity of SCZ and CMD (6). These evidences provide the**foundation for the genetic factors contribute to the comorbidity of SCZ and CMD. In this study, by utilizing several well-characterized methods of integrating the GWAS summary statistics of SCZ and CMD, and tissue-specific eQTL data and gene set database to translate the genetic risk loci into potential causal genes and pathways for them, we systematically predicted the candidate genes and biological pathways for SCZ and CMD. Through integrating the results from different approaches, we first revealed 21 potential pleiotropic genes and three biological pathways that are likely to be shared between SCZ and CMD.

Among the 21 potential pleiotropic genes, there are five genes associated with the comorbidity of SCZ and multiple phenotypes of CMD. *NT5C2* encodes a cytosolic purine 5′‐nucleotidase (cytosolic 5′‐nucleotidase II) involved in cellular purine metabolism (53), which is associated with SCZ, BMI, and CAD. Previous studies have shown that loss of function of *NT5C2* gene reduced body weight gain, improved glucose tolerance, reduced plasma insulin and triglyceride in high-fat diet mice (54). However, a recent study showed that knockdown of neuronal CG32549 in D. melanogaster, which is similar to NT5C2 protein in human, is associated with impaired motility behavior (55). These results strongly suggest that *NT5C2* may play a key role in SCZ and CMD. *C12orf65* is associated with SCZ, HDL, and T2D. The *C12orf65* gene encodes a mitochondrial matrix protein participating in the process of mitochondrial translation (56). Although multiple phenotypes have been shown to be associated with mutations in *C12orf65* gene, including early-onset optic atrophy, encephalomyopathy, peripheral neuropathy, intellectual disability, and spastic paraparesis (56–58), the function of *C12orf65* gene remains largely unknown. *SETD8* (*SET8*/*Pr-SET7*/*KMT5A*) is the causal gene for SCZ, HDL, and T2D. As a member of methyltransferase family specifically targeting Histone H4 Lys20 for methylation, *SETD8* plays an important role in cellular senescence, proliferation and apoptosis (59–61). Consistent with our results, multiple experiments indicated that changed expression level of *SETD8* may affect insulinoma cell proliferation (62), hyperglycemic memory (63), and lipid metabolism (64). One study has shown that reducing the expression levels of *SETD8* may contribute to altered hippocampal cellular composition, impaired neurodevelopment, and subsequent neurocognitive impairment (65), which are associated with the phenotype of SCZ. *GATAD2A* is a subunit of the nucleosome remodeling and histone deacetylase (NuRD) complex, which is generally associated with embryonic development, cellular differentiation, and the repression of transcription (66). Although our results showed that *GATAD2A* is associated with SCZ, T2D, TC, and TG, the pathogenesis is still unclear. Recent studies showed that NuRD complex is involved in neuronal development (67), cardiac and skeletal muscle structural and metabolic (68). These results indicated that *GATAD2A* may contribute to the comorbidity of SCZ and CMD. *TM6SF2* is associated with SCZ, TC, and TG. The *TM6SF2* gene encodes a multi-pass membrane protein localized in the endoplasmic reticulum and the ER-Golgi intermediate compartment (69). Several studies *in vivo* and *in vitro* have proved that *TM6SF2* is closely related to abnormal metabolism of blood lipids, especially plasma TC and TG (70, 71). However, the detailed mechanisms contributing to SCZ are still poorly understood.

Beyond these genes associated with the comorbidity of SCZ and multiple phenotypes of CMD, there are 16 potential pleiotropic genes associated with SCZ and one phenotype of CMD. In detail, *VRK2*, *INO80E*, *YPEL3*, and *MAPK3* are the common candidate genes for SCZ and BMI. *FES* and *FURIN* are the candidate genes for both SCZ and CAD. *ARL6IP4*, *OGFOD2*, *PITPNM2*, *CDK2AP1*, *ABCB9*, *MPHOSPH9*, *SREBF1*, and *TOM1L2* are associated with the comorbidity of SCZ and T2D. *SLC39A8* and *AMBRA1* are associated with the comorbidity of SCZ and HDL. Except that two genes (*FURIN* and *SREBF1*) have strongly suggested association with the phenotype of SCZ and CMD, the role of other genes in the pathogenesis of both SCZ and corresponding phenotype of CMD remains unknown. *FURIN* encodes a protein of the proprotein convertases family, which processes proproteins through limited proteolysis and convert them into bioactive proteins and peptides (72). Accumulating evidence suggests that *FURIN* plays a critical role in atherosclerosis through regulation of lipid metabolism and vascular inflammation (73). Recent studies also showed that overexpression of *FURIN* in monocyte/macrophage cell promoted migration, increased proliferation, and reduced apoptosis (74), which might contribute to atherogenesis. Intriguingly, studies of the function of *FURIN* in brain have shown that knockdown of *FURIN* decrease head size, and inhibit human neural progenitor cells migrate (35). Overexpression of *FURIN* enhances long-term potentiation and spatial learning and memory performance (75). Further study is needed to state the role of *FURIN* in SCZ and CAD. *SREBF1* is a transcription factor participates in lipogenesis (76), insulin resistance (77), and inflammatory response (78), which may contribute to the development of T2D. Intriguingly, a recent study showed that *SREBF1* is associated with multiple subphenotypes of SCZ, such as hyperlocomotor activity in dark, depression-like and aggressive behaviors, and social deficits (79). These evidences suggest that *SREBF1* is likely associated with the comorbidity of SCZ and CMD. Although most of these genes have been confirmed to be related to the occurrence and development of SCZ and CMD, there is no experiment to confirm whether they are related to the comorbidity of SCZ and CMD.

To identify the potential biological pathways for SCZ and CMD, we conducted KEGG pathway enrichment analysis for all significant causal genes of SCZ and CMD. The results show that dopaminergic synapse and adrenergic signaling in cardiomyocytes are likely shared between SCZ and BMI. However, when we performed KEGG pathway enrichment analysis for the potential candidate genes of SCZ and CMD, there is no significant pathway enrichment. This may be caused by the function of some candidate genes are still unclear. To further explore the potential biological pathways shared between SCZ and CMD, we performed gene set enrichment using GSA-SNP2 and MAGMA. Our results showed that MAPK-TRK pathway shared between SCZ and BMI, growth hormone signaling and regulation of insulin secretion signaling shared between SCZ and T2D. The MAPK-TRK pathway is mainly regulated by neurotrophic factor ligands (e.g. brain-derived neurotropic factor, nerve growth factor) binding to tropomyosin‐related kinase (Trk) receptor, which was associated with neuronal survival and morphogenesis, hippocampal long-term potentiation, and synaptic plasticity (80, 81). Loss of Trk signaling also has been linked with food intake regulation and body weight (82, 83). These evidences strongly suggest that the MAPK-TRK pathway may be related to the comorbidity of SCZ and BMI. The insulin and growth hormone signaling are closely related to the occurrence and development of T2D, and the biological effects of insulin and growth hormone are involved in lipid metabolism, carbohydrate metabolism, and glucose metabolism (84–86), which are potential therapeutic effectiveness for T2D. Intriguingly, a recent clinical research showed that insulin and growth hormone signaling were associated with the development of SCZ (87). Further research is worthwhile to explore the insulin and growth hormone signaling in the comorbidity of SCZ and CMD.

There were some limitations of the current study. First, the major samples in the GWAS summary datasets came from populations of European ancestry, and it is worthwhile to validate in other ethnic groups. Second, to generate highly credible candidate genes for the comorbidity of SCZ and CMD, the causal gene for a disease was chosen if it is predicated by two or more than two approaches. Although these genes are promising candidate genes for SCZ and CMD, genes supported by individual prediction approach may also have a role in disease. Third, the eQTL datasets used in this study mostly came from normal human tissues, which may miss the candidate gene for diseases. Lastly, though this study identified potential candidate genes shared between SCZ and CMD, further biological experiments are needed to demonstrate the role of these genes in the comorbidity of SCZ and CMD.

In summary, we first characterized the landscape of potential pleiotropic genes and biological pathways that are likely to be shared between SCZ and CMD. Through integrating the GWAS summary statistics, tissue-specific eQTL data and gene set database, we identified some potential candidate genes and biological pathways for SCZ and CMD (including BMI, CAD, T2D, HDL, LDL, TC, and TG), respectively. In total, we revealed 21 potential pleiotropic genes and three biological pathways shared between SCZ and CMD, which will enable us to better understand the etiology for the comorbidity of SCZ and CVD.

## Data Availability Statement

Publicly available datasets were analyzed in this study. The links to download GWAS datasets can be found in **Supplementary Table S1**. Other data including eQTL datasets and the code of software for data analysis could be obtained from the resource described in *Materials and Methods*.

## Author Contributions

HL contributed to data analysis and wrote the manuscript. YS and XZ were responsible for technical support and revised the manuscript. SL and DH contributed to data and method preparation. LX and YC contributed to plot pictures and tables. LH and DW were responsible for the study design and supervised the whole study. All authors read and approved the final manuscript.

## Funding

This work was funded by grants from by National Key R&D Program of China (NO. 2017YFC0909400) and NSFC project (No. 91439203).

## Conflict of Interest

The authors declare that the research was conducted in the absence of any commercial or financial relationships that could be construed as a potential conflict of interest.

## References

[1] Owen MJ Sawa A Mortensen PB Schizophrenia. Lancet (2016) 388:86–97. 10.1016/S0140-6736(15)01121-6 26777917 PMC4940219

[2] Westman J Eriksson SV Gissler M Hällgren J Prieto ML Bobo WV Increased cardiovascular mortality in people with schizophrenia: a 24-year national register study. Epidemiol Psychiatr Sci (2018) 27:519–27. 10.1017/S2045796017000166 PMC6137375 28580898

[3] Vancampfort D Knapen J Probst M van Winkel R Deckx S Maurissen K Considering a frame of reference for physical activity research related to the cardiometabolic risk profile in schizophrenia. Psychiatry Res (2010) 177:271–9. 10.1016/j.psychres.2010.03.011 20406713

[4] Correll CU Solmi M Veronese N Bortolato B Rosson S Santonastaso P Prevalence, incidence and mortality from cardiovascular disease in patients with pooled and specific severe mental illness: a large-scale meta-analysis of 3,211,768 patients and 113,383,368 controls. World Psychiatry (2017) 16:163–80. 10.1002/wps.20420 PMC5428179 28498599

[5] de Hert M CU C Bobes J Cetkovich-Bakmas M Cohen D Asai I Physical illness in patients with severe mental disorders. I. Prevalence, impact of medications and disparities in health care. World Psychiatry (2011) 10:52–77. 10.1002/j.2051-5545.2011.tb00014.x 21379357 PMC3048500

[6] Andreassen OA Djurovic S Thompson WK Schork AJ Kendler KS O’Donovan MC Improved detection of common variants associated with schizophrenia by leveraging pleiotropy with cardiovascular-disease risk factors. Am J Hum Genet (2013) 92:197–209. 10.1016/j.ajhg.2013.01.001 23375658 PMC3567279

[7] Postolache TT Del Bosque-Plata L Jabbour S Vergare M Wu R Gragnoli C Co-shared genetics and possible risk gene pathway partially explain the comorbidity of schizophrenia, major depressive disorder, type 2 diabetes, and metabolic syndrome. Am J Med Genet B Neuropsychiatr Genet (2019) 180:186–203. 10.1002/ajmg.b.32712 30729689 PMC6492942

[8] So HC Chau KL Ao FK Mo CH Sham PC Exploring shared genetic bases and causal relationships of schizophrenia and bipolar disorder with 28 cardiovascular and metabolic traits. Psychol Med (2019) 49:1286–98. 10.1017/S0033291718001812 30045777

[9] Pritchard JK Przeworski M Linkage disequilibrium in humans: models and data. Am J Hum Genet (2001) 69:1–14. 10.1086/321275 11410837 PMC1226024

[10] Wu Y Zeng J Zhang F Zhu Z Qi T Zheng Z Integrative analysis of omics summary data reveals putative mechanisms underlying complex traits. Nat Commun (2018) 9:918. 10.1038/s41467-018-03371-0 29500431 PMC5834629

[11] GTEx Consortium The Genotype-Tissue Expression (GTEx) pilot analysis: multitissue gene regulation in humans. Science (2015) 348:648–60. 10.1126/science.1262110 PMC4547484 25954001

[12] Consortium SW Ripke S Neale BM Corvin A Walters JT Farh K-H Biological insights from 108 schizophrenia-associated genetic loci. Nature (2014) 511:421–7. 10.1038/nature1359 PMC4112379 25056061

[13] Locke AE Kahali B Berndt SI Justice AE Pers TH Day FR Genetic studies of body mass index yield new insights for obesity biology. Nature (2015) 518:197–206. 10.1038/nature14177 25673413 PMC4382211

[14] Consortium TC Nikpay M Goel A Won HH Hall LM Willenborg C A comprehensive 1000 Genomes–based genome-wide association meta-analysis of coronary artery disease. Nat Genet (2015) 47:1121–30. 10.1038/ng.3396 PMC4589895 26343387

[15] Mahajan A Taliun D Thurner M Robertson NR Torres JM Rayner NW Fine-mapping type 2 diabetes loci to single-variant resolution using high-density imputation and islet-specific epigenome maps. Nat Genet (2018) 50:1505–13. 10.1038/s41588-018-0241-6 PMC6287706 30297969

[16] Teslovich TM Musunuru K Smith AV Edmondson AC Stylianou IM Koseki M Biological, clinical and population relevance of 95 loci for blood lipids. Nature (2010) 466:707–13. 10.1038/nature09270 PMC3039276 20686565

[17] Schadt EE Molony C Chudin E Hao K Yang X Lum PY Mapping the genetic architecture of gene expression in human liver. PloS Biol (2008) 6:e107. 10.1371/journal.pbio.0060107 18462017 PMC2365981

[18] Myers AJ Gibbs JR Webster JA Rohrer K Zhao A Marlowe L A survey of genetic human cortical gene expression. Nat Genet (2007) 39:1494–9. 10.1038/ng.2007.16 17982457

[19] Westra HJ Peters MJ Esko T Yaghootkar H Schurmann C Kettunen J Systematic identification of trans eQTLs as putative drivers of known disease associations. Nat Genet (2013) 45:1238–43. 10.1038/ng.2756 PMC3991562 24013639

[20] Lloyd-Jones LR Holloway A McRae A Yang J Small K Zhao J The genetic architecture of gene expression in peripheral blood. Am J Hum Genet (2017) 100:228–37. 10.1016/j.ajhg.2016.12.008 PMC5294670 28065468

[21] Qi T Wu Y Zeng J Zhang F Xue A Jiang L Identifying gene targets for brain-related traits using transcriptomic and methylomic data from blood. Nat Commun (2018) 9:2282. 10.1038/s41467-018-04558-1 29891976 PMC5995828

[22] Battle A Mostafavi S Zhu X Potash JB Weissman MM McCormick C Characterizing the genetic basis of transcriptome diversity through RNA-sequencing of 922 individuals. Genome Res (2014) 24:14–24. 10.1101/gr.155192.113 24092820 PMC3875855

[23] GTEx Consortium Genetic effects on gene expression across human tissues. Nature (2017) 550:204–13. 10.1038/nature24277 PMC5776756 29022597

[24] Watanabe K Taskesen E van Bochoven A Posthuma D Functional mapping and annotation of genetic associations with FUMA. Nat Commun (2017) 8:1826. 10.1038/s41467-017-01261-5 29184056 PMC5705698

[25] Kircher M Witten DM Jain P O’Roak BJ Cooper GM Shendure J A general framework for estimating the relative pathogenicity of human genetic variants. Nat Genet (2014) 46:310–5. 10.1038/ng.2892 PMC3992975 24487276

[26] He X Fuller CK Song Y Meng Q Zhang B Yang X Sherlock: detecting gene-disease associations by matching patterns of expression QTL and GWAS. Am J Hum Genet (2013) 92:667–80. 10.1016/j.ajhg.2013.03.022 PMC3644637 23643380

[27] Zhu Z Zhang F Hu H Bakshi A Robinson MR Powell JE Integration of summary data from GWAS and eQTL studies predicts complex trait gene targets. Nat Genet (2016) 48:481–7. 10.1038/ng.3538 27019110

[28] Hu Y Li M Lu Q Weng H Wang J Zekavat SM A statistical framework for cross-tissue transcriptome-wide association analysis. Nat Genet (2019) 51:568–76. 10.1038/s41588-019-0345-7 PMC6788740 30804563

[29] Mancuso N Freund MK Johnson R Shi H Kichaev G Gusev A Probabilistic fine-mapping of transcriptome-wide association studies. Nat Genet (2019) 51:675–82. 10.1038/s41588-019-0367-1 PMC6619422 30926970

[30] Stančáková A Civelek M Saleem NK Soininen P Kangas AJ Cederberg H Hyperglycemia and a common variant of GCKR are associated with the levels of eight amino acids in 9,369 Finnish men. Diabetes (2012) 61:1895–902. 10.2337/db11-1378 PMC3379649 22553379

[31] Stančáková A Javorský M Kuulasmaa T Haffner SM Kuusisto J Laakso M Changes in insulin sensitivity and insulin release in relation to glycemia and glucose tolerance in 6,414 Finnish men. Diabetes (2009) 58:1212–21. 10.2337/db08-1607 PMC2671053 19223598

[32] Wright FA Sullivan PF Brooks AI Zou F Sun W Xia K Heritability and genomics of gene expression in peripheral blood. Nat Genet (2014) 46:430–7. 10.1038/ng.2951 PMC4012342 24728292

[33] Nuotio J Oikonen M Magnussen CG Jokinen E Laitinen T Hutri-Kähönen N Cardiovascular risk factors in 2011 and secular trends since 2007: the cardiovascular risk in Young Finns Study. Scand J Public Health (2014) 42:563–71. 10.1177/1403494814541597 25053467

[34] Raitakari OT Juonala M Rönnemaa T Keltikangas-Järvinen L Räsänen L Pietikäinen M Cohort profile: the cardiovascular risk in Young Finns Study. Int J Epidemiol. (2008) 37:1220–6. 10.1093/ije/dym225 18263651

[35] Fromer M Roussos P Sieberts SK Johnson JS Kavanagh DH Perumal TM Gene expression elucidates functional impact of polygenic risk for schizophrenia. Nat Neurosci (2016) 19:1442–53. 10.1038/nn.4399 PMC5083142 27668389

[36] Pers TH Karjalainen JM Chan Y Westra HJ Wood AR Yang J Biological interpretation of genome-wide association studies using predicted gene functions. Nat Commun (2015) 6:5890. 10.1038/ncomms6890 25597830 PMC4420238

[37] Pers TH Timshel P Ripke S Lent S Consortium SW Sullivan PF Comprehensive analysis of schizophrenia-associated loci highlights ion channel pathways and biologically plausible candidate causal genes. Hum Mol Genet (2016) 25:1247–54. 10.1093/hmg/ddw007 PMC4764200 26755824

[38] Li Z Chen J Yu H He L Xu Y Zhang D Genome-wide association analysis identifies 30 new susceptibility loci for schizophrenia. Nat Genet (2017) 49:1576–83. 10.1038/ng.3973 28991256

[39] Bentley AR Sung YJ Brown MR Winkler TW Kraja AT Ntalla I Multi-ancestry genome-wide gene–smoking interaction study of 387,272 individuals identifies new loci associated with serum lipids. Nat Genet (2019) 51:636–48. 10.1038/s41588-019-0378-y PMC6467258 30926973

[40] Scott RA Scott LJ Mägi R Marullo L Gaulton KJ Kaakinen M An expanded genome-wide association study of type 2 diabetes in Europeans. Diabetes (2017) 66:2888–902. 10.2337/db16-1253 PMC5652602 28566273

[41] Võsa U Claringbould A Westra HJ Bonder MJ Deelen P Zeng B Unraveling the polygenic architecture of complex traits using blood eQTL metaanalysis. bioRxiv (2018), [Preprint]. 10.1101/447367

[42] Yu G Wang LG Han Y He QY clusterProfiler: an R package for comparing biological themes among gene clusters. OMICS (2012) 16:284–7. 10.1089/omi.2011.0118 PMC3339379 22455463

[43] Yoon S Nguyen HC Yoo YJ Kim J Baik B Kim S Efficient pathway enrichment and network analysis of GWAS summary data using GSA-SNP2. Nucleic Acids Res (2018) 46:e60. 10.1093/nar/gky175 29562348 PMC6007455

[44] de Leeuw CA Mooij JM Heskes T Posthuma D MAGMA: generalized gene-set analysis of GWAS data. PloS Comput Biol (2015) 11:e1004219. 10.1371/journal.pcbi.1004219 25885710 PMC4401657

[45] 1000 Genomes Project Consortium Auton A GR A DM A RM D GR A A global reference for human genetic variation. Nature (2015) 526:68–74. 10.1038/nature15393 26432245 PMC4750478

[46] Hauberg ME Zhang W Giambartolomei C Franzén O Morris DL Vyse TJ Large-scale identification of common trait and disease variants affecting gene expression. Am J Hum Genet (2017) 100:885–94. 10.1016/j.ajhg.2017.04.016 PMC5474225 28552197

[47] Willer CJ Speliotes EK Loos RJF Li S Lindgren CM Heid IM Six new loci associated with body mass index highlight a neuronal influence on body weight regulation. Nat Genet (2009) 41:25–34. 10.1038/ng.287 19079261 PMC2695662

[48] Liu R Zou Y Hong J Cao M Cui B Zhang H Rare loss-of-function variants in NPC1 predispose to human obesity. Diabetes (2017) 66:935–47. 10.2337/db16-0877 28130309

[49] Hackinger S Prins B Mamakou V Zengini E Marouli E Brčić L Evidence for genetic contribution to the increased risk of type 2 diabetes in schizophrenia. Transl Psychiatry (2018) 8:252. 10.1038/s41398-018-0304-6 30470734 PMC6251918

[50] Krzywinski M Schein J Birol I Connors J Gascoyne R Horsman D Circos: an information aesthetic for comparative genomics. Genome Res (2009) 19:1639–45. 10.1101/gr.092759.109 PMC2752132 19541911

[51] Amare AT Schubert KO Klingler-Hoffmann M Cohen-Woods S Baune BT The genetic overlap between mood disorders and cardiometabolic diseases: a systematic review of genome wide and candidate gene studies. Transl Psychiatry (2017) 7:e1007. 10.1038/tp.2016.261 28117839 PMC5545727

[52] Burmeister M McInnis MG Zöllner S Psychiatric genetics: progress amid controversy. Nat Rev Genet (2008) 9:527–40. 10.1038/nrg2381 18560438

[53] Oka J Matsumoto A Hosokawa Y Inoue S Molecular cloning of human cytosolic purine 5′-nucleotidase. Biochem Biophys Res Commun (1994) 205:917–22. 10.1006/bbrc.1994.2752 7999131

[54] Johanns M Kviklyte S Chuang SJ Corbeels K Jacobs R Herinckx G Genetic deletion of soluble 5′-nucleotidase II reduces body weight gain and insulin resistance induced by a high-fat diet. Mol Genet Metab (2019) 126:377–87. 10.1016/j.ymgme.2019.01.017 30803894

[55] Duarte RRR Bachtel ND Côtel M-C Lee SH Selvackadunco S Watson IA The psychiatric risk gene NT5C2 regulates adenosine monophosphate-activated protein kinase signaling and protein translation in human neural progenitor cells. Biol Psychiatry (2019) 86:120–30. 10.1016/j.biopsych.2019.03.977 PMC6614717 31097295

[56] Antonicka H Østergaard E Sasarman F Weraarpachai W Wibrand F Pedersen AM Mutations in C12orf65 in patients with encephalomyopathy and a mitochondrial translation defect. Am J Hum Genet (2010) 87:115–22. 10.1016/j.ajhg.2010.06.004 PMC2896764 20598281

[57] Buchert R Uebe S Radwan F Tawamie H Issa S Shimazaki H Mutations in the mitochondrial gene C12ORF65 lead to syndromic autosomal recessive intellectual disability and show genotype phenotype correlation. Eur J Med Genet (2013) 56:599–602. 10.1016/j.ejmg.2013.09.010 24080142

[58] Spiegel R Mandel H Saada A Lerer I Burger A Shaag A Delineation of C12orf65-related phenotypes: a genotype–phenotype relationship. Eur J Hum Genet (2014) 22:1019–25. 10.1038/ejhg.2013.284 PMC4350599 24424123

[59] Tanaka H Takebayashi Si Sakamoto A Igata T Nakatsu Y Saitoh N The SETD8/PR-Set7 methyltransferase functions as a barrier to prevent senescence-associated metabolic remodeling. Cell Rep (2017) 18:2148–61. 10.1016/j.celrep.2017.02.021 28249161

[60] Dhami GK Liu H Galka M Voss C Wei R Muranko K Dynamic methylation of Numb by Set8 regulates its binding to p53 and Apoptosis. Mol Cell (2013) 50:565–76. 10.1016/j.molcel.2013.04.028 23706821

[61] Abbas T Shibata E Park J Jha S Karnani N Dutta A CRL4^Cdt2^ regulates cell proliferation and histone gene expression by targeting PR-Set7/Set8 for degradation. Mol Cell (2010) 40:9–21. 10.1016/j.molcel.2010.09.014 20932471 PMC2966975

[62] Li Z Liu H Niu Z Zhong W Xue M Wang J Temporal proteomic analysis of pancreatic β-cells in response to lipotoxicity and glucolipotoxicity. Mol Cell Proteomics (2018) 17:2119–131. 10.1074/mcp.RA118.000698 PMC6210228 30082485

[63] Chen X Wu Q Jiang H Wang J Zhao Y Xu Y SET8 is involved in the regulation of hyperglycemic memory in human umbilical endothelial cells. Acta Biochim Biophys Sin (Shanghai) (2018) 50:635–42. 10.1093/abbs/gmy051 29762637

[64] Liao T Wang YJ Hu JQ Wang Y Han LT Ma B Histone methyltransferase KMT5A gene modulates oncogenesis and lipid metabolism of papillary thyroid cancer in vitro. Oncol Rep (2018) 39:2185–92. 10.3892/or.2018.6295 29512765

[65] Ke X Xing B Yu B Yu X Majnik A Cohen S IUGR disrupts the PPARγ-Setd8-H4K20me1 and Wnt signaling pathways in the juvenile rat hippocampus. Int J Dev Neurosci (2014) 38:59–67. 10.1016/j.ijdevneu.2014.07.008 25107645 PMC4268161

[66] Torchy MP Hamiche A Klaholz BP Structure and function insights into the NuRD chromatin remodeling complex. Cell Mol Life Sci (2015) 72:2491–507. 10.1007/s00018-015-1880-8 PMC11114056 25796366

[67] Nitarska J Smith JG Sherlock WT Hillege MMG Nott A Barshop WD A functional switch of NuRD chromatin remodeling complex subunits regulates mouse cortical development. Cell Rep (2016) 17:1683–98. 10.1016/j.celrep.2016.10.022 PMC5149529 27806305

[68] Gómez-del Arco P Perdiguero E Yunes-Leites PS Acín-Pérez R Zeini M Garcia-Gomez A The chromatin remodeling complex Chd4/NuRD controls striated muscle identity and metabolic homeostasis. Cell Metab (2016) 23:881–92. 10.1016/j.cmet.2016.04.008 27166947

[69] Mahdessian H Taxiarchis A Popov S Silveira A Franco-Cereceda A Hamsten A TM6SF2 is a regulator of liver fat metabolism influencing triglyceride secretion and hepatic lipid droplet content. Proc Natl Acad Sci U S A (2014) 111:8913–8. 10.1073/pnas.1323785111 PMC4066487 24927523

[70] Smagris E Gilyard S BasuRay S Cohen JC Hobbs HH Inactivation of Tm6sf2, a gene defective in fatty liver disease, impairs lipidation but not secretion of very low density lipoproteins. J Biol Chem (2016) 291:10659–76. 10.1074/jbc.M116.719955 PMC4865914 27013658

[71] Holmen OL Zhang H Fan Y Hovelson DH Schmidt EM Zhou W Systematic evaluation of coding variation identifies a candidate causal variant in TM6SF2 influencing total cholesterol and myocardial infarction risk. Nat Genet (2014) 46:345–51. 10.1038/ng.2926 PMC4169222 24633158

[72] Jaaks P Bernasconi M The proprotein convertase furin in tumour progression. Int J Cancer (2017) 141:654–63. 10.1002/ijc.30714 28369813

[73] Ren K Jiang T Zheng XL Zhao GJ Proprotein convertase furin/PCSK3 and atherosclerosis: new insights and potential therapeutic targets. Atherosclerosis (2017) 262:163–70. 10.1016/j.atherosclerosis.2017.04.005 28400053

[74] Zhao G Yang W Wu J Chen B Yang X Chen J Influence of a coronary artery disease–associated genetic variant on FURIN expression and effect of Furin on macrophage behavior. Arterioscler Thromb Vasc Biol (2018) 38:1837–44. 10.1161/ATVBAHA.118.311030 PMC6092112 29976768

[75] Zhu B Zhao L Luo D Xu D Tan T Dong Z Furin promotes dendritic morphogenesis and learning and memory in transgenic mice. Cell Mol Life Sci (2018) 75:2473–88. 10.1007/s00018-017-2742-3 PMC11105492 29302702

[76] Takeuchi Y Yahagi N Aita Y Murayama Y Sawada Y Piao X KLF15 enables rapid switching between lipogenesis and gluconeogenesis during fasting. Cell Rep (2016) 16:2373–86. 10.1016/j.celrep.2016.07.069 PMC5031553 27545894

[77] Bi Y Wu W Shi J Liang H Yin W Chen Y Role for sterol regulatory element binding protein-1c activation in mediating skeletal muscle insulin resistance via repression of rat insulin receptor substrate-1 transcription. Diabetologia (2014) 57:592–602. 10.1007/s00125-013-3136-1 24362725

[78] Oishi Y Spann NJ Link VM Muse ED Strid T Edillor C SREBP1 contributes to resolution of pro-inflammatory TLR4 signaling by reprogramming fatty acid metabolism. Cell Metab (2017) 25:412–27. 10.1016/j.cmet.2016.11.009 PMC5568699 28041958

[79] Lee S Kang S Ang MJ Kim J Kim JC Kim SH Deficiency of sterol regulatory element-binding protein-1c induces schizophrenia-like behavior in mice. Genes Brain Behav (2019) 18:e12540. 10.1111/gbb.12540 30430717

[80] Pandya CD Kutiyanawalla A Pillai A BDNF–TrkB signaling and neuroprotection in schizophrenia. Asian J Psychiatr (2013) 6:22–8. 10.1016/j.ajp.2012.08.010 PMC3565158 23380313

[81] Minichiello L Calella AM Medina DL Bonhoeffer T Klein R Korte M Mechanism of TrkB-mediated hippocampal long-term potentiation. Neuron (2002) 36:121–37. 10.1016/S0896-6273(02)00942-X 12367511

[82] Rosas-Vargas H Martínez-Ezquerro JD Bienvenu T Brain-derived neurotrophic factor, food intake regulation, and obesity. Arch Med Res (2011) 42:482–94. 10.1016/j.arcmed.2011.09.005 21945389

[83] Mason BL Lobo MK Parada LF Lutter M Trk B signaling in dopamine 1 receptor neurons regulates food intake and body weight. Obesity (Silver Spring) (2013) 21:2372–6. 10.1002/oby.20382 PMC3742719 23512795

[84] Fridlyand LE Tamarina NA Schally AV Philipson LH Growth hormone-releasing hormone in diabetes. Front Endocrinol (2016) 7:129. 10.3389/fendo.2016.00129 PMC5056186 27777568

[85] Liu Z Cordoba-Chacon J Kineman RD Cronstein BN Muzumdar R Gong Z Growth hormone control of hepatic lipid metabolism. Diabetes (2016) 65:3598–609. 10.2337/db16-0649 PMC5127251 27679560

[86] List EO Palmer AJ Berryman DE Bower B Kelder B Kopchick JJ Growth hormone improves body composition, fasting blood glucose, glucose tolerance and liver triacylglycerol in a mouse model of diet-induced obesity and type 2 diabetes. Diabetologia (2009) 52:1647–55. 10.1007/s00125-009-1402-z 19468705

[87] van Beveren NJ Schwarz E Noll R Guest PC Meijer C de Haan L Evidence for disturbed insulin and growth hormone signaling as potential risk factors in the development of schizophrenia. Transl Psychiatry (2014) 4:e430. 10.1038/tp.2014.5 25158005 PMC4150237

